# Analysis of asymptomatic and clinical malaria in urban and suburban settings of southwestern Ethiopia in the context of sustaining malaria control and approaching elimination

**DOI:** 10.1186/s12936-016-1298-2

**Published:** 2016-04-30

**Authors:** Guofa Zhou, Delenasaw Yewhalaw, Eugenia Lo, Daibin Zhong, Xiaoming Wang, Teshome Degefa, Endalew Zemene, Ming-chieh Lee, Estifanos Kebede, Kora Tushune, Guiyun Yan

**Affiliations:** Program in Public Health, University of California, Irvine, CA 92617 USA; Department of Medical Laboratory Sciences and Pathology, College of Health Sciences, Jimma University, Jimma, Ethiopia; Tropical and Infectious Diseases Research Center, Jimma University, Jimma, Ethiopia; Department of Health Management, College of Health Sciences, Jimma University, Jimma, Ethiopia

**Keywords:** Malaria, Incidence, Prevalence, Risk factor, Urban area, Ethiopia

## Abstract

**Background:**

Malaria intervention in Ethiopia has been strengthened significantly in the past decade. The Ethiopian government recently stratified the country based upon annual parasite incidence into malaria free, low, moderate and high transmission strata. Districts with low transmission were targeted for indigenous transmission elimination. Surveillance on malaria disease incidence is needed for planning control and elimination efforts.

**Methods:**

Clinical malaria was monitored prospectively in health facilities in Jimma town, Oromia Region, southwestern Ethiopia from July 2014 to June 2015. Seasonal cross-sectional parasite prevalence surveys in local communities were conducted in 2014 and 2015 in eight *kebeles*. Case report forms were administered to obtain sociodemographic and epidemiological information from patients.

**Results:**

A total of 1434 suspected malaria cases were examined from the health facilities and 428 confirmed malaria cases were found. Among them, 327 (76.4 %) cases were *Plasmodium vivax*, 97 (22.7 %) were *Plasmodium falciparum*, and 4 (0.9 %) were mixed infection of *P. vivax* and *P. falciparum*. The annual malaria incidence rate was 1.7 cases per 1000 people at risk. Parasite prevalence in the community was less than 3 %. Household ownership of insecticide-treated nets (ITNs) was 47.3 % (1173/2479) and ITN usage was 37.9 %. All ITNs were long-lasting insecticidal nets, and repellent use was not found in the study area. Being male and traveling were the significant risk factors for *P. falciparum* malaria. For *P. vivax* malaria, risk factors included occupation and history of malaria illness during the preceding 30 days.

**Conclusion:**

Epidemiological evidence suggested low clinical malaria incidence and prevalence in Jimma town. More aggressive measures may be needed to further suppress vivax transmission. Strategies should be planned targeting sustained control and elimination.

**Electronic supplementary material:**

The online version of this article (doi:10.1186/s12936-016-1298-2) contains supplementary material, which is available to authorized users.

## Background

Ethiopia is one of the few countries in Africa where *Plasmodium vivax* and *Plasmodium falciparum* coexist [1, 2]. It is also one of the few African countries with a policy of providing malaria prevention and control services including malaria diagnosis and treatment, insecticide-treated bed nets (ITNs), and indoor residual spray (IRS) free of charge [3–6]. This policy ensures that malaria interventions reach the poor, and in turn enables them to increase economic productivity [4]. In Ethiopia, major malaria intervention scale-up efforts began in 2004/2005 with the introduction of artemisinin-based combination therapy (ACT) as the first-line treatment of *P. falciparum* malaria. ACT has been free of charge for all ages in the public sector since then [3–5]. IRS campaigns have targeted epidemic-prone areas, and IRS coverage has reached to 37 % of the at-risk population in 2013 [4–6]. Long-lasting insecticidal nets (LLINs) have been distributed free of charge to the entire population through mass campaigns to step up vector control and prevention since 2006 [3, 4]. In 2008, the Ethiopian government distributed 20.4 million LLINs, and 11.2 million were replaced in 2010 and 7 million in 2011 [3, 4]. The combined improvement in coverage of essential interventions has coincided with a considerable reduction in malaria morbidity and mortality as well as epidemic episodes and affected areas. While no epidemics have been reported in the country since 2004 [7], a comprehensive longitudinal study showed that malaria remains a major public health problem in Ethiopia [8, 9].

The latest National Malaria Strategic Plan of Ethiopia stratified districts into malaria free, low, moderate and high transmission areas based upon annual parasite incidence [4, 10]. An ambitious goal was set to eliminate indigenous transmission in low transmission areas [4, 10]. In some districts where malaria control is shifted to elimination, changes have been made in the malaria treatment guideline. For example, a single low-dose primaquine is added to the artemether-lumefantrine (Coartem^®^) treatment regimen for *P. falciparum* to kill gametocytes and reduce malaria transmission. A 14-day course of primaquine is added to the chloroquine treatment regimen for radical cure of *P. vivax* malaria in low transmission area [10]. In the past 10 years, IRS has been performed on an annual basis and LLINs have been distributed to all residents in Jimma town free of charge [4]. However, data on the current coverage of interventions (e.g., LLIN ownership and population coverage), epidemiological characteristics (e.g., age and gender distribution) and clinical malaria risk factors broadly in Ethiopia as well as in local areas are still lacking.

The present study determined the current epidemiological characteristics of malaria in urban and suburban sites of southwestern Ethiopia. Prospective passive case detection was conducted in health centers and district hospitals to determine clinical malaria incidence in parallel with cross-sectional surveys to determine parasite prevalence in the community. This information on malaria epidemiology can inform malaria control and elimination strategies in Ethiopia.

## Methods

### Study area

**Fig. 1:**
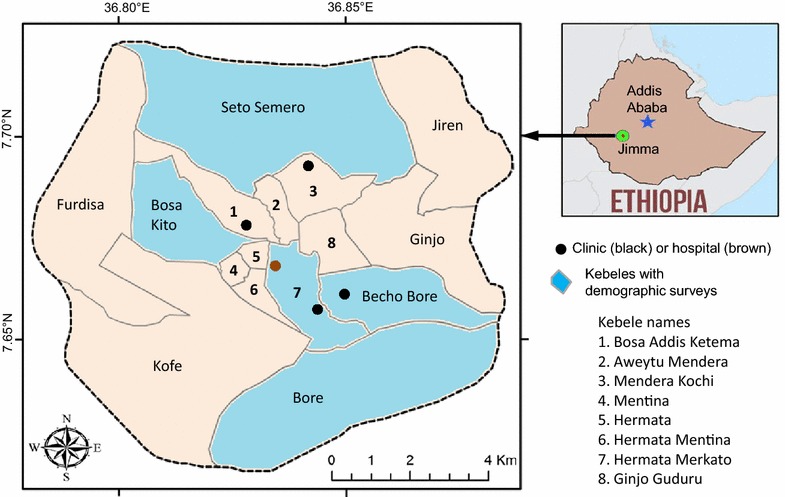
Catchment area, locations of health facilities and localities where demographic information was surveyed in Jimma town, southwestern Ethiopia

The study was conducted in Jimma town (7°41′ N, 36°50′ E, 1710–1800 m above sea level) in a highland low-transmission epidemic-prone area in Oromia Region, southwestern Ethiopia (Fig. 1). The population of Jimma town was about 176,500 in 2015. Jimma town has a warm climate with a mean annual temperature of 23 °C, a mean annual maximum temperature of 27 °C, and a mean annual minimum temperature of 19 °C. The mean annual precipitation is about 1500 mm, with one rainy season from May to September. December and January are the driest months.

### Passive malaria case detection (PCD)

**Fig. 2:**
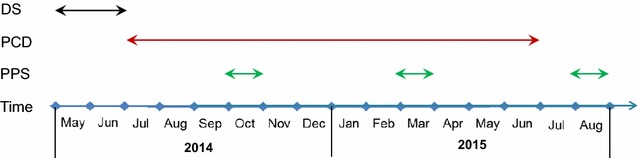
Chronology of the study: demographic survey, cross-sectional parasite prevalence survey and passive case surveillance

PCD was conducted prospectively in all public health centres and hospitals in Jimma town including Becho Bore Health Centre, Jimma Health Centre, Jimma Higher Two Health Centre, Mendera Kochi Health Centre, and Shenen Gibe Hospital, except Jimma University Special Hospital (Fig. 1). Jimma University Special Hospital usually accepts referred severe malaria cases, to avoid double count, malaria cases from this hospital were not included in data analysis. These are all the government-run malaria treatment centres and hospitals. A clinical malaria case is defined as an individual with malaria-related symptoms (fever, i.e., axillary temperature ≥37.5 °C, chills, severe malaise, headache or vomiting) at the time of examination or 1–2 days prior to the examination, with a *Plasmodium* positive blood smear [11, 12]. All febrile patients with malaria-like symptoms attending these health facilities were enrolled into this study upon the signing of consent or assent forms for minors. Standard case report forms were used to collect the following information from each patient: village of residency, demographic characteristics, occupation, education level, clinical symptoms, blood hemoglobin level, history of malaria in the preceding 30 days, history of travel in the 2 weeks preceding the clinic visit, history of fever, treatment drug prescribed, and ownership and use of malaria prevention measures. If ITN was used, the types and usage (used or not during the preceding night) of ITN were recorded. Thick and thin blood smears were prepared for each patient. Slides were stained with 10 % Giemsa and examined microscopically by experienced laboratory technologists at the Medical Parasitology Laboratory of Jimma University. Species were identified and parasites were counted against 200 leukocytes. All positive slides and 10 % of negative slides were reexamined blindly by another senior laboratory technologist. PCD was conducted from July 2014 to June 2015 (Fig. 2).

### Cross-sectional parasitological survey

Cross-sectional parasite prevalence surveys were conducted three times in October 2014 (high-transmission season), March 2015 (end of high-transmission season), and August 2015 (end of low-transmission season), in eight *kebeles* (the smallest administrative unit in Ethiopia, equivalent to a district in a city) representing both urban and suburban settings (Figs. 1, 2). Households were randomly selected from each *kebele* to maximize coverage so that the surveyed population would represent the eight *kebeles*. To avoid sampling bias, one volunteer was arbitrarily selected from each selected household for participation of this study. Peripheral blood was collected by the standard finger-prick method using disposable lancets in October 2014, March and August 2015, respectively. Thick and thin smears were prepared. Slides were stained and examined at the Jimma University Specialized Hospital laboratory according to standard World Health Organization procedures. Smears were stained with Giemsa solution, and examined microscopically at a magnification of 100× by experienced laboratory technologists. Parasitaemia was determined, and quality control for slide reading was implemented as described above. Each study participant found positive for malaria was referred to a nearby health facility for treatment, according to the national malaria diagnosis and treatment guidelines.

A subset of the blood samples was subjected to further PCR analysis to confirm parasite infection and to identify the parasite species [13–15]. The initially plan was to do PCR on all blood samples, due to the zero prevalence rate detected by microscopy, PCR was only done on randomly selected samples after the second round survey.

Sample size was estimated based on the binomial model. Because the prevalence of malaria was low in the area, an estimate of 10 % prevalence was used for the peak-transmission season and 5 % for the low-transmission season in sample size determination [13, 16]. The resulting sample sizes were 559 persons for the peak-transmission season and 386 for the low-transmission season for prevalence estimation at precision level of 5 %. For the PCR method, assuming ≤5 % prevalence based on microscopic results, with a Type I error of 5 % and population (blood samples) size of 850, 218 samples could detect a marginal error of 2.5 %. To allow for 10 % PCR failure rate, 240 samples is enough.

### Demographic survey

The demographic survey was conducted in the same eight *kebeles* as the parasitological surveys (Figs. 1, 2). Households were randomly selected from each *kebele* to maximize coverage so that the surveyed population would represent the eight *kebeles*. All individuals living in the selected households were included in the study. For all consenting individuals, demographic characteristics (age, sex, occupation, and education level) and information on preventive measures (ITN, IRS, repellent, and others) were collected through questionnaires. The survey was conducted from May to June 2014. Information collected on ITNs included ITN ownership, type, and usage. The total population at each *kebeles* in Jimma was obtained from the Central Statistical Agency of Ethiopia based on 2015 demographic projections. Household ITN ownership was calculated as percentage of households that owns at least one ITN based on demographic survey.

### Data analysis

The density of parasitaemia was expressed as the number of asexual *Plasmodium* per microlitre of blood, assuming a leukocyte count of 8000 per microlitre. Annual malaria incidence rate was calculated as the number of cases per 1000 population at risk. Parasite prevalence rate was calculated as the number of infected individuals per 100 people. The incidence rate was stratified into three age groups: age < 5, 5 ≤ age < 15, and ≥15 years. Differences in sex-, age-, and occupation-specific incidence rates were analysed using χ^2^ test and odds ratios (ORs) were calculated for different categories separately against their respective reference group. Risk factors of clinical malaria were analysed using multivariate nominal logistic regression analysis. Risk factors included age, sex, occupation, history of malaria in the preceding 30 days, history of travel in the preceding 2 weeks, and preventive measure usage. Risk factor analysis was done separately for *P. falciparum* and *P. vivax*. SPSS (IBM Corporation, Armonk, New York, USA) was used for data analysis.

### Ethical statement

Ethical clearance was obtained from the Institutional Review Boards (IRBs) of Jimma University, Ethiopia, and the University of California at Irvine, USA. Written consent (assent for children under 18 years) was obtained from heads of households and study participants. All cases with a history of fever in the preceding 3 days, as well as those with fever on examination and a positive test for malaria parasite in the blood film examination, were offered anti-malarial treatment by physicians from Jimma University as per national malaria diagnosis and treatment guidelines. Infants younger than 6 months and individuals who were unwilling to participate were excluded from the study.

## Results

### Epidemiological characteristics of clinical malaria

A total of 1434 suspected cases were examined and clinical malaria was confirmed in 428 cases. Among the confirmed cases, 327 (76.4 %) were *P. vivax*, 97 (22.7 %) were *P. falciparum*, and four (0.9 %) were mixed infections of *P. vivax* and *P. falciparum*. No *Plasmodium malariae* or *Plasmodium ovale* was detected. Demographic information was obtained from 10,119 individuals comprising 5278 (52.2 %) females and 4841 (47.8 %) males, with an average age of 20.7 years (Additional file 1). Among the confirmed malaria cases, 299 resided within the eight villages where demographic information was collected and available. Among these cases, 224 (74.9 %) were *P. vivax*, 71 (23.7 %) were *P. falciparum*, and 4 (1.3 %) were mixed infections of *P. vivax* and *P. falciparum*. The annual malaria incidence rate was 1.7 cases per 1000 people at risk (Table 1).

**Table 1:** Epidemiological characteristics of clinical malaria in Jimma town, southwestern Ethiopia

Parameter	Population^a^	*P. vivax*		*P. falciparum*	
		Incidence rate	Odds ratio (95 % CI)	Incidence rate	Odds ratio (95 % CI)
Overall	176,498	1.27		0.40	
Gender					
Female (%)	52.16	1.33	1	0.30	1
Male (%)	47.84	1.21	0.91 [0.70, 1.19]	0.51	1.68 [1.04, 2.71]*
Age (year)					
<5 (%)	14.64	1.32	1	0.46	1
≥5 and <15 (%)	25.04	1.45	1.10 [0.72, 1.68]	0.43	0.92 [0.45, 1.91]
≥15 (%)	60.32	1.18	0.90 [0.61, 1.32]	0.38	0.81 [0.42, 1.54]
Occupation					
Trader/traveler (%)	10.90	0.53	1	0.32	1
Non-schooled child (%)^b^	18.95	0.85	1.62 [0.78, 3.35]	0.27	0.86 [0.31, 2.43]
Students (%)^c^	29.82	1.22	2.33 [1.19, 4.56]*	0.44	1.44 [0.57, 3.46]
Housewife (%)	15.37	1.42	2.73 [1.36, 5.52]**	0.41	1.30 [0.48, 3.53]
Farmer and worker (%)^d^	22.15	1.72	3.32 [1.70, 6.48]***	0.55	1.73 [0.70, 4.30]
Officer and Teacher (%)	2.80	3.92	7.88 [3.62, 17.15]***	0.21	0.65 [0.08, 5.41]

*, ** and *** represent significance at level of 0.05, 0.01 and 0.001, respectively

^a^Based on 2013 population and percentage was calculated based on the 2014 demographic surveys of 10,119 individuals

^b^Non-schooled children includes all children (<15 years) who are not attending any regular school

^c^Includes all students from kindergarten to college students

^d^Farmer, factory worker, construction worker, gardener, casual worker, and unemployed

**Fig. 3:**
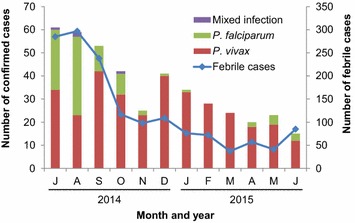
Monthly dynamics of number of case report forms and confirmed clinical malaria cases by parasite species in Jimma town, southwestern Ethiopia

Total number of case report forms and confirmed malaria cases decreased monotonously from July 2014 to June 2015 (Fig. 3). *Plasmodium falciparum* malaria was seasonal and was mainly observed from July to October 2014, but the seasonality in clinical *P*. *vivax* malaria was not obvious (Fig. 3). While males had significantly higher *P. falciparum* malaria incidence rates (OR = 1.68, 95 % CI [1.04, 2.71], P < 0.05) (Table 1) than females, there was no gender difference in *P. vivax* malaria. Incidence rates did not vary by age for either malaria species (Table 1). There was a significant difference in *P. vivax* incidence rates among different occupation groups (Table 1). Trader and travellers had the lowest *P. vivax* incidence rate (0.53 cases per 1000 people year), whereas office workers and teachers had the highest incidence rate compared to the other groups (3.92 cases per 1000 people year; OR = 3.39, 95 % CI [2.08, 5.50]). Although incidence rates of *P. falciparum* malaria varied substantially among occupation groups, the variation was not statistically significant due to small number of falciparum cases detected (Table 1).

Five categories of malaria preventive measures were included in the questionnaire: bed nets, indoor residual spraying (IRS), repellent, others, and none. The household ITN ownership rate (percentage of households that owns at least one ITN) was 47.3 % (1173/2479) and ITN usage rate (i.e., proportion of individuals who reported to use the net the night before the survey) was 37.5 %. All ITNs were LLINs, and no repellent use was found in the questionnaire survey. The use of LLIN was not significantly associated with clinical malaria risk (Table 2). Travel during the preceding 14 days did not affect *P. vivax* malaria incidence, but it increased the likelihood of *P. falciparum* malaria by fourfolds (OR = 4.14, 95 % CI [2.11, 8.14], P < 0.001) (Table 2). On the other hand, having malaria during the preceding 30 days increased the likelihood of *P. vivax* malaria by nearly threefolds (OR = 2.63, 95 % CI [1.51, 4.57], P < 0.001) (Table 2).

**Table 2:** Univariate analysis of risk factors of clinical malaria in Jimma town, south western Ethiopia

Risk factor	Case reported	*P. vivax*		*P. falciparum*	
		Cases^a^	Odds ratio (95 %CI)	Cases^a^	Odds ratio (95 % CI)
Use of prevention measure					
Yes	661	152	1	47	1
No	773	175	0.98 [0.77, 1.26]	50	0.90 [0.60, 1.36]
Use of ITN					
Used ITN	609	199	1	59	1
Not available or not use	825	131	1.16 [0.90, 1.49]	37	1.19 [0.78, 1.82]
Travel during the preceding 14 days					
No, not traveled	1378	311	1	85	1
Yes, traveled	56	16	1.37 [0.76, 2.48]	12	4.14 [2.11, 8.14]***
Malaria during the preceding 30 days					
No, no malaria	1380	304	1	91	1
Yes, had malaria	54	23	2.63 [1.51, 4.57]***	6	1.77 [0.74, 4.25]

*^,^**^,^ *** represent significant level of 0.05, 0.01 and 0.001, respectively

^a^Confirmed cases

Multiple logistic regression analysis indicated that male had marginally higher risk than females, and that people who travelled during the preceding 14 days had a significantly higher risk of having *P. falciparum* malaria after adjusting for other risk factors (Additional file 2). For *P. vivax* malaria, being an office worker or teacher, or had malaria during the preceding 30 days were the significant risk factors (Additional file 3).

### Parasite prevalence in cross-sectional survey

In the cross-sectional survey low parasite prevalence was found consistently among the three sampling time points (Table 3). Overall, parasite prevalence was 0.8 % by microscope and 2.4 % by PCR. Microscopically identified parasite prevalence was about 2.7 % (0.8 % *P. falciparum* and 1.9 % *P. vivax*) during the high-transmission season in October 2014. Unfortunately, PCR was not done for this survey due to the loss of blood samples during sample transportation. Microscopy did not identify any infection for the surveys conducted in March and August 2015, but PCR analysis detected a prevalence of 2.6 and 1.7 %, respectively (Table 3). Overall, there were 23 (56.1 %) *P. vivax* positive samples and 18 (43.9 %) *P. falciparum* positive samples in the three surveys. No *P. malariae*, *P. ovale*, or mixed infections were identified by either microscopy or PCR.

**Table 3:** Microscopy- and PCR-based parasite prevalence of cross-sectional surveys in Jimma town, south western Ethiopia

Collection date	Microscopy (%)			Nested PCR (%)		
	Sample size	*Pf*	*Pv*	Sample size	*Pf*	*Pv*
October, 2014	622	0.8	1.9	Not done		
March, 2015	755	0	0	727	1.2	1.4
August, 2015	833	0	0	290	1.4	0.3

*Pf* *Plasmodium falciparum*; *Pv* *Plasmodium vivax*

## Discussion

With the support of the Global Fund to Fight AIDS, Tuberculosis and Malaria, the President’s Malaria Initiative and from the African national governments, there has been a massive scale-up of antimalarial interventions in the past decade. These interventions have led to wide-scale reductions in malaria morbidity and mortality and have changed the landscape of malaria epidemiology in Africa. For example, many studies have shown that household ITN ownership has reached more than 80 % in Eastern Africa [17–19] and that the scaling-up of malaria control has greatly reduced malaria-related burdens [20–26]. Despite high coverage of ITNs and IRS, some areas, however, showed a resurgence of malaria in recent years [27–32]. This phenomenon could be linked to strong insecticide resistance in *Anopheles* mosquitoes [33–35]. Compare to findings from a previous study, parasite prevalence rate in Jimma has been reduced by more than 50 % in the last 5 years (5.2 % in 2010 vs. 2.5 % in 2015). While it is a significant reduction in malaria prevalence, parasite species compositions are comparable between the two studies [8].

In this study, household ITN ownership in Jimma town was 47 % and usage was 38 %. These percentages were close to the national average [5] but far below the government target of universal coverage of at-risk populations by 2015 [4]. ITN users in the study area had a slightly lower but insignificant risk of clinical malaria compared to those who did not use ITNs. This coincided with earlier reports that suggested ITN use did not reduce *P. falciparum* prevalence [16, 32, 36]. The ineffectiveness of ITNs may be attributed to factors such as insecticide resistance [37–39], early biting and outdoor transmission [16, 29, 36]. In Jimma, *Anopheles arabiensis* is the major malaria vector [16, 37, 40, 41]. Previous blood-meal analysis in *An. arabiensis* in Ethiopia found that the human blood index (HBI) and bovine blood index (BBI) were similar; i.e., the vector fed almost equally on human and animals [37, 40]. This implies that even if ITN usage by humans is 100 %, indoor prevention measures such as ITNs and IRS do not protect people who rest and work outdoor [37, 39, 40, 42].

Changes in age and gender distribution represent a common epidemiological shift in malaria-eliminating countries, where increasing proportions of adults and males were observed among all malaria cases regardless of malaria parasite species [43, 44]. In this study, males in the study area had higher clinical *P. falciparum* malaria incidence rates than females, consistent with previous studies [8, 45]. The reason for this is unclear, but it is possible that males may spend longer time outdoor during evening and thus expose more frequent to mosquito biting. Age is a malaria risk factor especially in areas of high transmission intensity [37, 43, 45–47]. In high-transmission settings, young children typically have the highest parasite prevalence and clinical incidence [12, 48–50], whereas in areas of very low transmission both children and adults are vulnerable groups [43, 44]. The finding that age was not a significant risk factor of malaria prevalence is consistent with the epidemiological situation in low transmission areas.

It is not surprising that a history of malaria illness in the preceding 30 days posed a risk for *P. vivax* malaria but not *P*. *falciparum* malaria. This was likely due to the relapse of *vivax* parasite arising from persistent liver stages of hypnozoites [51]. In the tropics, *P*. *vivax* relapse is a common phenomenon. This finding has critical implications in guiding malaria control and elimination in Ethiopia. For *P*. *falciparum* malaria, it seems that the current front-line treatment policy, e.g., first-line treatment with ACT, is possibly effective and sufficient for the elimination of falciparum malaria, given the continuous decline in clinical falciparum malaria incidence [1]. By contrast, clinical *P. vivax* malaria remains unchanged in the study area. More aggressive treatment such as radical cure with primaquine may be needed to avoid relapse of vivax malaria. However, it is noteworthy that extreme caution must be taken when implement primaquine for radical cure of vivax malaria because primaquine may cause acute hemolysis in individuals with Glucose-6-phosphate dehydrogenase (G6PD) deficiency, although Such effect may be less severe or absent if dosage was low [52].

It is interesting that occupation was found to be a risk factor for *P. vivax* malaria but not for *P*. *falciparum* malaria in the present study. Indoor workers such as teachers and office workers showed a four-fold higher risk of getting *vivax* malaria than outdoor workers. A possible explanation could be differential treatment-seeking behavior as the office workers and teachers are financially capable of seeking health care at the health centres [9]. Although anti-malarial treatment is free in Ethiopia, all patients are required to pay clinical registration (~US $1.00) and laboratory test (~US $0.25 per test) fees. On the other hand, individuals who travelled in the preceding 14 days showed a high risk of getting falciparum malaria. This likely resulted from being exposed to *P. falciparum* in surrounding rural areas with high transmission intensity [43, 44].

In this study, *P*. *falciparum* malaria was seasonal, however, seasonality in *P*. *vivax* malaria was not clear. The high number of clinical vivax cases from July to September in 2014 was inconsistent with entomological observations and rainfall patterns in the area, where the peak transmission season was expected to be from October to December [16]. Therefore, the declining trend in clinical malaria from July 2014 to June 2015 may be an overall declining in malaria transmission in the area, but yet needs to be confirmed with long-term observations.

The limitation of this study is that malaria cases were collected only from government-run malaria treatment centres and hospitals of the study areas. By the government policy, sales of anti-malarial drugs by private shops are prohibited, so self-treatment of malaria in Ethiopia is very unlikely. Malaria cases could have been underestimated because patients may seek treatments in other small private clinics that were not included here. Also, another major hospital, Jimma University Special Hospital that is specialized in treating severe malaria was not included. Although the number of malaria cases is known to be small and some case may have been reported from elsewhere, this may slightly influence the estimations of this study.

## Conclusion

The urbanization process results in profound socioeconomic and landscape changes that generally reduce malaria transmission [53, 54]. However, population movement between urban areas and surrounding malarious rural areas presents an important risk to residents in urban areas. In Ethiopia, where *P. falciparum* and *P. vivax* coexist, the effects of urbanization and human movement on the clinical disease incidence of these two species were not the same, and should be investigated further. Clinical malaria disease incidence and parasite prevalence data in Jimma town suggest very low transmission that could be targeted for elimination. Epidemiological studies and risk factor analysis suggest malaria control and elimination in urban settings where *P. falciparum* and *P. vivax* coexist require new strategies.

## Additional files

10.1186/s12936-016-1298-2 Demographic characteristics of the study population.
10.1186/s12936-016-1298-2 Results of multiple regression. Dependent variable was *Plasmodium falciparum* (confirmed positive or negative).
10.1186/s12936-016-1298-2 Results of multiple regression. Dependent variable was *Plasmodium vivax* (confirmed positive or negative).
