# Supplementary material for: Analysis of asymptomatic and clinical malaria in urban and suburban settings of southwestern Ethiopia in the context of sustaining malaria control and approaching elimination
Source: Malar J. 2016 Apr 30;15:250. doi: 10.1186/s12936-016-1298-2 (PMC4851815; doi:10.1186/s12936-016-1298-2)
Supplement: Supplementary file 1 — 10.1186/s12936-016-1298-2 Demographic characteristics of the study population. [file 12936_2016_1298_MOESM1_ESM.docx]

Additional file Table S1. Demographic characteristics of the study population

| Demographic characteristic | | Population | Percentage |
| --- | --- | --- | --- |
| Total surveyed | | 10,119 |  |
| Sex |  |  |  |
|  | Female | 5,278 | 52.2 |
|  | Male | 4,841 | 47.8 |
| Age (years) | |  |  |
|  | 0~4 | 1,481 | 14.6 |
|  | 5~14 | 2,534 | 25.0 |
|  | >=15 | 6,104 | 60.3 |
| Education level | |  |  |
|  | None † | 3,277 | 32.4 |
|  | Primary | 5,198 | 51.4 |
|  | Middle/high school | 1,383 | 13.7 |
|  | College and above | 261 | 2.6 |
| Occupation | |  |  |
|  | Students | 2,968 | 29.9 |
|  | Non-schooled children | 1,886 | 19.0 |
|  | Housewife | 1,530 | 15.4 |
|  | Casual worker | 1,351 | 13.6 |
|  | Trader | 1,083 | 10.9 |
|  | Unemployed | 523 | 5.3 |
|  | Farmer, outdoor worker | 307 | 3.1 |
|  | Officer worker, soldier, teacher | 278 | 2.8 |
| ITN ownership and usage | |  |  |
|  | No ITN | 5,193 | 51.3 |
|  | Own ITN but not using it | 1,086 | 10.7 |
|  | Used ITN | 3,840 | 37.9 |

† includes illiteracy adults and non-schooled children.
