# Supplementary material for: Analysis of asymptomatic and clinical malaria in urban and suburban settings of southwestern Ethiopia in the context of sustaining malaria control and approaching elimination
Source: Malar J. 2016 Apr 30;15:250. doi: 10.1186/s12936-016-1298-2 (PMC4851815; doi:10.1186/s12936-016-1298-2)
Supplement: Supplementary file 2 — 10.1186/s12936-016-1298-2 Results of multiple regression. Dependent variable was Plasmodium falciparum (confirmed positive or negative). [file 12936_2016_1298_MOESM2_ESM.docx]

Additional file 2

Table S2. Results of multiple regression. Dependent variable was *P. falciparum* (confirmed positive or negative)

| Term | Coefficient | ChiSquare | Prob>ChiSq | Odds Ratio (95% CI) |
| --- | --- | --- | --- | --- |
| Intercept | 1.95 | 28.19 | <.0001 | n.a. |
| Age [<5 yrs] | -0.16 | 0.20 | 0.6536 | 0.73 [0.19, 2.97] |
| Age [5 to <15 yrs] | -0.06 | 0.05 | 0.8190 | 0.88 [0.32, 2.76] |
| Sex [Male] | 0.25 | 3.82 | 0.0507 | 1.65 [1.01, 2.78] |
| Occupation [Farmer, outdoor worker]† | 0.02 | 0.00 | 0.9482 | 1.04 [0.35, 3.05] |
| Occupation [Trader and traveler] | -0.06 | 0.03 | 0.8554 | 0.88 [0.24, 3.57] |
| Occupation [Officer and teacher] | 0.97 | 2.03 | 0.1544 | 6.96 [0.68, 181.84] |
| Occupation [Students] ^§^ | 0.04 | 0.02 | 0.8869 | 1.09 [0.34, 3.58] |
| Occupation [Housewife] | -0.21 | 0.37 | 0.5442 | 0.66 [0.17, 2.55] |
| Education [Illiteracy] | 0.41 | 1.26 | 0.2614 | 2.29 [0.57, 10.48] |
| Education [Primary school] | 0.08 | 0.10 | 0.7578 | 1.18 [0.42, 3.35] |
| Education [Middle school] | 0.03 | 0.01 | 0.9209 | 1.07 [0.30, 4.18] |
| Education [High school] | 0.00 | 0.00 | 0.9959 | 1.00 [0.20, 6.28] |
| Malaria during the preceding 30 days [Yes] | 0.32 | 2.19 | 0.1386 | 1.90 [0.75, 4.20] |
| Travel during the preceding 14 days [Yes] | 0.51 | 6.79 | 0.0092 | 2.80 [1.22, 5.82] |
| ITN use [Yes] | -0.09 | 0.58 | 0.4451 | 0.84 [0.54, 1.30] |

^†^ Also includes factory worker, construction worker, gardener, casual worker, and unemployed.

^§^ Includes all students from kindergarten to college students.
