# Supplementary material for: Analysis of asymptomatic and clinical malaria in urban and suburban settings of southwestern Ethiopia in the context of sustaining malaria control and approaching elimination
Source: Malar J. 2016 Apr 30;15:250. doi: 10.1186/s12936-016-1298-2 (PMC4851815; doi:10.1186/s12936-016-1298-2)
Supplement: Supplementary file 3 — 10.1186/s12936-016-1298-2 Results of multiple regression. Dependent variable was Plasmodium vivax (confirmed positive or negative). [file 12936_2016_1298_MOESM3_ESM.docx]

Additional file 3

Table S3. Results of multiple regression. Dependent variable was *P. vivax* (confirmed positive or negative)

| Term | Coefficient | ChiSquare | Prob>ChiSq | Odds Ratio (95% CI) |
| --- | --- | --- | --- | --- |
| Intercept | 0.95 | 13.44 | 0.0002 | n.a. |
| Age [<5 yrs] | 0.22 | 1.08 | 0.2978 | 1.57 [0.67, 3.64] |
| Age [5 to <15 yrs] | -0.04 | 0.06 | 0.8061 | 0.92 [0.46, 1.77] |
| Sex [Male] | -0.02 | 0.06 | 0.8095 | 0.97 [0.71, 1.29] |
| Occupation [Farmer, outdoor worker]† | 0.02 | 0.01 | 0.9226 | 1.03 [0.53, 2.02] |
| Occupation [Trader and traveler] | 0.14 | 0.43 | 0.5142 | 1.31 [0.57, 2.95] |
| Occupation [Officer and teacher] | 0.75 | 6.55 | 0.0105 | 4.46 [1.40, 13.97] |
| Occupation [Students] ^§^ | 0.04 | 0.04 | 0.8383 | 1.08 [0.52, 2.23] |
| Occupation [Housewife] | -0.10 | 0.24 | 0.6240 | 0.82 [0.10, 1.81] |
| Education [Illiteracy] | 0.30 | 1.87 | 0.1713 | 1.83 [0.78, 4.45] |
| Education [Primary school] | 0.16 | 0.93 | 0.3352 | 1.38 [0.72, 2.71] |
| Education [Middle school] | 0.29 | 1.81 | 0.1788 | 1.77 [0.78, 4.17] |
| Education [High school] | 0.13 | 0.24 | 0.6243 | 1.30 [0.47, 3.81] |
| Malaria during the preceding 30 days [Yes] | 0.46 | 9.87 | 0.0017 | 2.51 [1.40, 4.43] |
| Travel during the preceding 14 days [Yes] | -0.15 | 0.73 | 0.3935 | 0.75 [0.37, 1.42] |
| ITN use [Yes] | -0.09 | 1.88 | 0.1708 | 0.83 [0.64, 1.08] |

^†^ Also includes factory worker, construction worker, gardener, casual worker, and unemployed.

^§^ Includes all students from kindergarten to college students.
